# Caput medusae sign; a unique finding during abdominal examination in patients with portal hypertension; case report

**DOI:** 10.1016/j.amsu.2020.04.004

**Published:** 2020-04-24

**Authors:** Ayad Ahmad Mohammed

**Affiliations:** Department of Surgery, College of Medicine, University of Duhok, Kurdistan Region, Iraq

**Keywords:** Portal hypertension, Porto-systemic venous anastomosis, Variceal bleeding, Caput medusae, Hematemesis

## Abstract

Portal hypertension is an increase in the portal venous pressure resulting in the formation of dilated veins at the site of porto-systemic venous anastomosis causing shifting of the blood flow from the portal venous system to the systemic circulation.

A 53-year-old male presented to the emergency department complaining from hematemesis. He was admitted to the emergency department. Abdominal examination showed hugely dilated veins in the abdominal wall with palpable spleen and liver. The hemoglobin level was low and liver enzymes were mildly elevated. The patient received two units of blood and four units of fresh frozen plasma, intravenous propranolol and intravenous vasopressin. Endoscopy showed variceal bleeding which was mild, multiple bandings were performed for the bleeding vessels. The past medical history was negative apart from idiopathic portal vein thrombosis. He was on regular anticoagulants and beta blockers. The patient was prepared to undergo surgical shunting procedure.

Acute variceal bleeding is a medical emergency, and patients need aggressive form of treatment. Most drugs like beta-blockers, derivatives of vasopressin and somatostatins work by inducing splanchnic vasoconstriction and decrease the portal venous pressure. Endoscopic band ligation may be required but this has no effect on the portal venous pressure, other alternatives include trans-jugular intrahepatic portosystemic shunts or surgery.

## Highlights

• Caput medusae sign is a clinical sign indicating portal hypertension.
• It results from shifting of blood flow from the portal venous system to the systemic circulation.
• This results in the formation of dilated veins at the site of porto-systemic venous anastomosis.
• Portal hypertension results in splenomegaly, ascites, esophageal and gastric varices.

## 1 Introduction

Portal hypertension is defined as increase in the portal venous pressure resulting the formation of dilated veins at the site of porto-systemic venous anastomosis causing shifting of the blood flow from the portal venous system to the systemic circulation. Increase in the portal venous resistance is the initial factor in portal hypertension ([1]).

Other factors that are associated with portal hypertension are increase in the splanchnic blood flow due to splanchnic arterial dilatation and hyperdynamic circulation ([2]).

Portal hypertension is suggested clinically by the presence of splenomegaly, ascites, and the presence of dilated d veins in the abdominal wall and esophageal and gastric varices ([3]).

The presence of esophageal varices is found to be related directly with the severity of the underlying cause ([4]).

Patients may be asymptomatic when the varices are small and the liver function is maintained, or patients may present with acute variceal bleeding, the source of the bleeding may be either esophageal or gastric varices, gastric erosions are another main cause of bleeding ([5]).

Portal hypertension is associated with other complications such as ascites, hepatic encephalopathy and hepato-renal syndrome. The development of these complications is associated with high rate of morbidity and mortality ([3]).

Bleeding from the esophageal and the gastric variceal vessels will result in worsening of the portal hypertension by stimulating of various vasoactive gut substances and hormones such as glucagon and vasoactive intestinal polypeptide, this is a normal response to the presence of proteins in the bowel lumen which will lead to dilatation of the splanchnic vessels and increased in the portal blood flow and subsequently elevation in the portal venous pressure ([3]).

The work in this case report has been reported in line with the SCARE 2018 criteria ([6]).

## 2 Patient information

### 2.1 Clinical findings

A 53-year-old male, who is a known case of portal hypertension due to portal vein thrombosis, presented to the emergency department complaining from two attacks of hematemesis.

The patient was diagnosed as a case of idiopathic portal vein thrombosis before 4 years. He had repeated admissions to the hospital because of similar attacks of hematemesis which were managed conservatively. He had no history of alcohol consumption and he is smoker for the last 25 years. He was on regular anticoagulants and beta blockers. The past surgical history was negative.

**Fig. 1:**
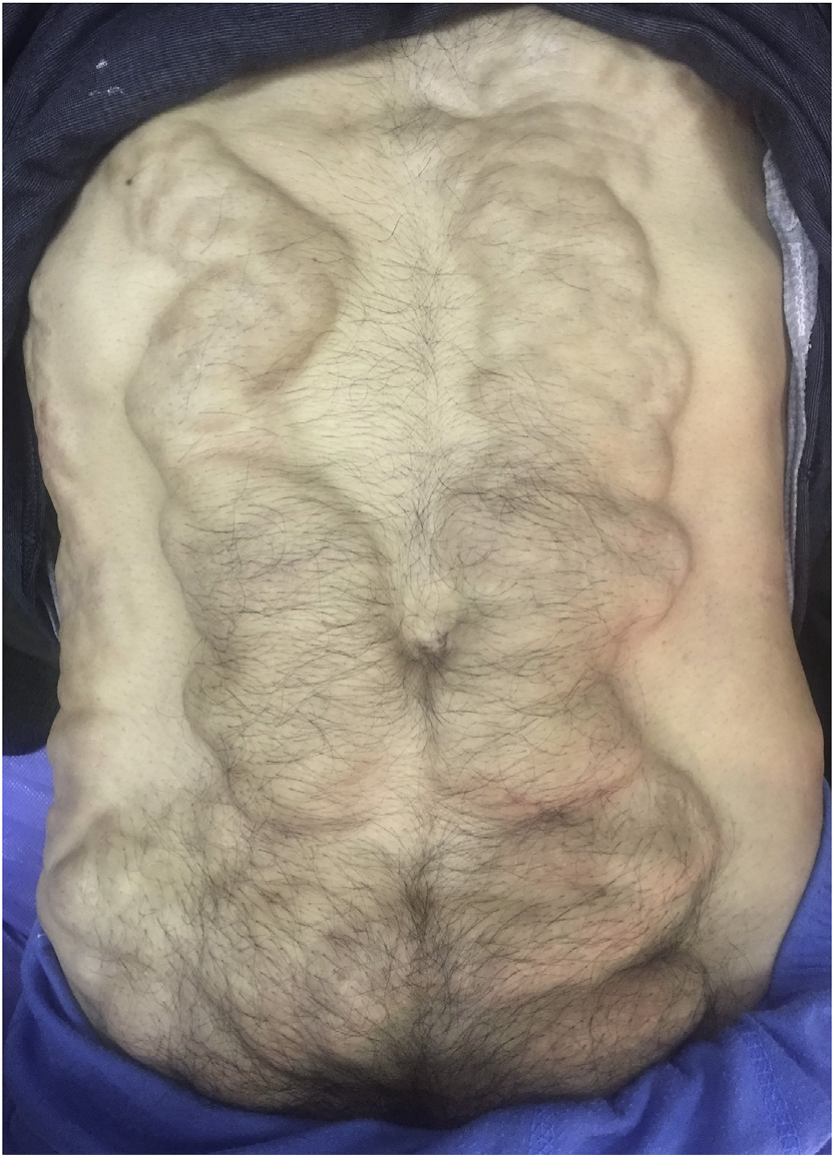
Hugely dilated veins in the abdominal wall and the flanks seen during abdominal examination.

Abdominal examination showed hugely dilated veins in the abdominal wall and the flanks, Fig. 1.

The spleen was palpable about 5 cm below the costal margin, and the liver was palpable 2 cm below the costal margin (liver span: 16 cm). There were no signs of shifting dullness for ascites.

He was admitted to the emergency department in Duhok Emergency Teaching Hospital (which serves a population of around one million people), the blood pressure was 105/65 mmHg, and the pulse rate was 100 beats/minute.

### 2.2 Diagnostic assessment

The investigations showed low hemoglobin level (9 G/L) and the liver enzymes were mildly elevated. The serum albumin was 3 g/L. The viral hepatitis profile was negative.

### 2.3 Therapeutic intervention

Preparation of compatible blood was done and he received two units of blood, with four units of fresh frozen plasma, intravenous propranolol and intravenous vasopressin were slowly given.

The patient developed no further attacks of hematemesis. He was sent for endoscopy which showed mild variceal bleeding, multiple bandings were performed for the bleeding vessels. Ultrasound showed mild ascites with hepatomegaly.

The patient was then admitted to the high dependency unit for one day and he was discharged home after 4 days.

No specific post-management considerations were undertaken.

### 2.4 Follow-up and outcomes

Follow up was done for 2 months later and he was prepared to undergo surgical shunting procedure.

## 3 Discussion

From the time of diagnosis of esophageal and gastric varices, patients have 25% risk to develop bleeding within the first 2 years. The high portal venous pressure can be modified by pharmacological agents and humeral substances such as glucagon, prostacyclin, endotoxins, and nitric oxide which, these will help to decrease the portal venous pressure by inducing splanchnic vasodilatation and causing reduction in the peripheral vasodilatation ([1,2]).

Patients who presented with emergency bleeding should be resuscitated until their general condition is stabilized, when endoscopic attempts fail to stop the bleeding or when the bleeding is severe, Sengstaken-Blakemore tube can be inserted and the gastric and esophageal balloon inflated for up to 24 hours. The patient should be monitored in the intensive care unit ([3]).

Most drugs which are used in patients with portal hypertension work by inducing splanchnic vasoconstriction and hence they have a role in decreasing the portal venous pressure. These drugs may include beta-blockers, derivatives of vasopressin and somatostatins ([2]).

Sodium restriction and diuretics help also to aid reduction in the systemic and portal venous pressure ([1]).

Hepatic venous pressure gradient may predict the development of future complications such as variceal bleeding, re-bleeding, and help to arrange follow-up intervals ([2]).

Acute variceal bleeding is a medical emergency, and patients need aggressive form of treatment, this may include resuscitation, medical therapy and endoscopic band ligation but this has no effect on the portal venous pressure. No more than 2 sessions of endoscopic band ligations should be performed; other alternatives may include trans-jugular intrahepatic porto-systemic shunts or surgery when other options failed, the last two options have direct effect to lower the portal venous blood flow and the portal pressure, and thus they decrease the rate of complications such as ascites, hepatic encephalopathy, and hepato-renal syndrome([2]).

Caput medusae sign is frequently mentioned in medical books as a sign of portal hypertension but such hugely dilated veins are very rarely seen in clinical practice and it is a unique finding during general and abdominal examination.

## Funding source

This research did not receive any funding from any resource.

## Author contribution

Study design, writing, and the final approval of the manuscript: Dr Ayad Ahmad Mohammed

## Guarantor

Dr Ayad Ahmad Mohammed

## Provenance and peer review

Not commissioned, externally peer reviewed.

## Patient perspective

I had this condition before and I was informed that when I developed bloody vomiting and black stool I should visit urgently the hospital.

## Informed consent

Written informed consent was obtained from the patient for publication of this case report and accompanying images.

## Declaration of competing interest

There is no conflict of interest.

## Appendix A Supplementary data

Application Application
